# An Account of an Abscess of the Mediastinum, Mistaken for an Aneurism

**Published:** 1781-12

**Authors:** 


### SECTION II.

### ESSAYS AND OBSERVATIONS.

I. *An account of an abſceſs of the Mediaſtinum, miſtaken for an Aneuriſm. By an ingenious Surgeon and teacher of Anatomy lately deceaſed. Communicated by a member of the Society. Read* December 10, 1781.

IT has been obſerved, that a ſuppuration ſometimes takes place between the two laminæ of the pleura that compoſe the mediaſtinum, and in ſuch caſes we are adviſed by M. de la Martiniere*, in his ingenious Memoir on this ſubject, to trepan the ſternum. But a diſeaſe of this kind occurs ſo rarely, that ſome practitioners of great knowledge and experience have doubted

* Mem. de l'Acad. de Chirurgie, Tome IV.

the the poſſibility of its exiſtence. It is certain, however, that ſeveral caſes of this ſort have happened, and I remember about the year 1758 to have ſeen an inſtance of it in a ſtrong, middle-aged man, by occupation a porter, who, in carrying a heavy burden along the ſtreet, fell with his breaſt againſt the pavement.

He was admitted into an hoſpital in London, and complained of great pain about the ſternum, the integuments of which were much bruiſed, but no fracture either of the ſternum or ribs could be diſcovered by the moſt accurate examination.

Venæſection, gentle evacuations by ſtool, a cooling regimen, and other means were employed to obviate any inflammatory ſymptoms that might ariſe. The patient complained of pain and difficulty in breathing, and in a few days after his admiſſion a tumour began to appear about the middle of the ſternum, the part that had been injured by the fall. This ſwelling increaſed by degrees to a conſiderable bulk, and was attended with a pulſation, like that of an aneuriſm, which was eaſily to be felt by placing a finger on each ſide of the ſternum.

Theſe appearances induced the ſurgeons to treat the caſe as an aneuriſm of the aorta. Venæſection næſection was frequently repeated, and the patient was directed to be kept quiet. The integuments, however, became gradually thinner and thinner, and at length burſt, but inſtead of an extravaſation of arterial blood and the death of the patient, both which were expected to take place, a great quantity of pus was diſcharged from the opening. The nature of the caſe being now underſtood, the wound was dilated, and the man obtained a cure.

It is obſervable, that in M. de la Martiniere's paper juſt now referred to, not a ſingle caſe is related in which the trepan was applied to the ſternum before the burſting of the tumour, and indeed we have as yet no characterizing ſymptoms laid down by which we can with certainty be enabled to diſtinguiſh this from ſome other diſeaſes; as the pain, difficulty of breathing, and ſyncope that are ſaid to attend it will likewiſe accompany a fracture of the ſternum, and in the caſe juſt now related there was no particular ſymptom preſent that could lead us to diſtinguiſh it from an aneuriſm of the aorta.

